# The earliest large carpenter bee (*Xylocopa*) and its adhering pollen (Araliaceae, Theaceae)

**DOI:** 10.1007/s12549-024-00604-7

**Published:** 2024-05-23

**Authors:** Christian Geier, Michael S. Engel, Johannes M. Bouchal, Silvia Ulrich, Friðgeir Grímsson, Sonja Wedmann, Torsten Wappler

**Affiliations:** 1 University of Vienna, Department of Botany and Biodiversity Research, Division of Structural and Functional Botany, Rennweg 14, 1030 Vienna, Austria; 2 American Museum of Natural History, Division of Invertebrate Zoology, 200 Central Park West, New York, NY 10024-5192 U.S.A.; 3 Universidad Nacional Mayor de San Marcos, Facultad de Ciencias Biológicas y Departamento de Entomología, Museo de Historia Natural, Av. Arenales 1256, Jesús María, Lima 14, Perú; 4 Museum at Prairiefire, 5801 West 135th Street, Overland Park, KS 66223 U.S.A.; 5 Austrian Academy of Sciences (OeAW), Austrian Archaeological Institute (OeAI), Department of Historical Archaeology, Dominikanerbastei 16, 1010 Vienna, Austria; 6 Senckenberg Forschungsinstitut und Naturmuseum Frankfurt Senckenberg Forschungsstation Grube Messel, Markstraße 35, 64409 Messel, Germany; 7 Hessisches Landesmuseum Darmstadt, Department of Natural History, Friedensplatz 1, 64283 Darmstadt, Germany; 8 Rheinische Friedrich-Wilhelms Universität Bonn, Institute of Geoscience, Paleontology Section, Nußallee 8, 53115 Bonn, Germany

**Keywords:** Eocene, Eudicots, Hymenoptera, Messel, Plant-insect interactions, Pollination

## Abstract

The association of pollinators with their host plants is a critical element of ecosystem functioning and one that is usually determined indirectly in the fossil record from specific morphological traits of flowers or putative pollinating animals. The exceptionally fine preservation at Messel, Germany, offers an excellent source of data on pollen from fossil flowers as well as preserved adhering to insects as direct evidence of their association with specific floral lineages. Here, we report on pollen recovered from the body and legs of a large carpenter bee (Apidae: Xylocopinae: Xylocopini) from the Eocene of Messel. The fossil is the earliest occurrence of the tribe Xylocopini and represents an extinct subgenus and species, described as *Xylocopa* (*Apocolyx*) *primigenia* subgen. et sp. nov. Two eudicot pollen types were recovered from the bee, one of the family Theaceae (Asterids: Ericales) and another of Araliaceae (Euasterids: Apiales). The pollen grains are compared with various extinct and extant pollen types, and data on floral visitors to modern theaceous and araliaceous flowers are explored in relation to understanding the association of the fossil carpenter with these floral types in the paratropical Eocene biota of Messel.

## Introduction

One of the most influential biological associations is the interrelationship between insects and plants. The evolutionary interplay between insects and plants, either as friends or foes, has shaped terrestrial ecosystems for at least the last 400 million years (Grimaldi and Engel 2005; Engel 2023). Of the myriad ways in which these titans of biodiversity interact, none is more intimate than that of pollination, a mutualism, with some specialised exceptions, that has aided the proliferation of both lineages (Grimaldi 1999; Engel 2023; Peña-Kairath et al. 2023; Peris and Condamine 2024). Indeed, today insects are the primary pollinators for nearly 90 % of all flowering plants (Ollerton et al. 2011). Perhaps among all of these insect pollinators, none are more iconic than the bees, a specialised lineage of phytophagous stinging wasps that feed their larvae pollen and nectar (Grimaldi and Engel 2005; Michener 2007; Engel et al. 2021). With over 20,500 species, bees are critical to the success of many terrestrial ecosystems through their pollination services and especially vital for agriculture and food security where there is a particular overreliance on honey bees (genus *Apis* Linnaeus). Over the last 120 million years, bees have evolved many specialisations for the gathering and processing of pollen, nectar, floral oils, and plant exudates (Almeida et al. 2023). While most bees are polylectic, numerous lineages have evolved various degrees of host-plant specialisation (oligolecty) and, in rare instances, outright monolecty (Michener 2007; Rasmussen et al. 2020; Engel et al. 2021). While much intensity is devoted to the study of bee pollination, an understanding of the evolutionary history of this association has been restricted mainly to indirect inferences either through phylogenetic estimation or from isolated fossil occurrences. For example, the inference of orchid pollination in Miocene Hispaniola based on the presence of orchid bees in Dominican amber even in the absence of attached orchid pollinia (Engel 1999). The history of insect pollination as documented by the fossil record is gaining momentum (Peña-Kairath et al. 2023), particularly through the exploration of pollen grains preserved on or within insect fossils belonging to pollinating lineages (e.g. Ramírez et al. 2007; Wappler et al. 2015; Grímsson et al. 2017; Wedmann et al. 2021; Geier et al. 2022; Geier et al. 2023a, b, c, d) and this has been especially true for the bees. Some of these exceptional fossils provide direct evidence of pollen collected by the bees during life and a glimpse into ancient insect-plant interactions (e.g. Ramírez et al. 2007; Wappler et al. 2015; Grímsson et al. 2017; Geier et al. 2023a).

Here, we report the discovery of a large carpenter bee (genus *Xylocopa* Latreille) from the middle Eocene of Messel, Germany. Species of *Xylocopa* have been previously reported as compression fossils from various localities, nearly all of which are from the Miocene (Engel 2001a; Michez et al. 2012). A notable exception was the fragmentary *Xylocopa gabrielae* Engel from the Eocene-Oligocene boundary of Florissant, Colorado (Engel 2001a), hitherto the earliest occurrence of the genus. The new species reported here now supersedes *X. gabrielae* in age. Of significance, the new fossil has adhering pollen preserved on the body, allowing for an investigation into the floral visits of this individual immediately prior to death and providing a snapshot of bee-flower relationships during the Eocene. Direct evidence of floral visitation by fossil insects is exceptionally limited. We describe the xylocopine bee and its adhering pollen and explore the occurrences of the floral families represented and their modern insect visitors in relation to the discovery of their association with the bee documented herein. This is the first documentation of pollen from a fossil carpenter bee and only the third genus of bees from the Eocene in which adhering pollen has been recorded.

## Material and methods

**Fig. 1:**
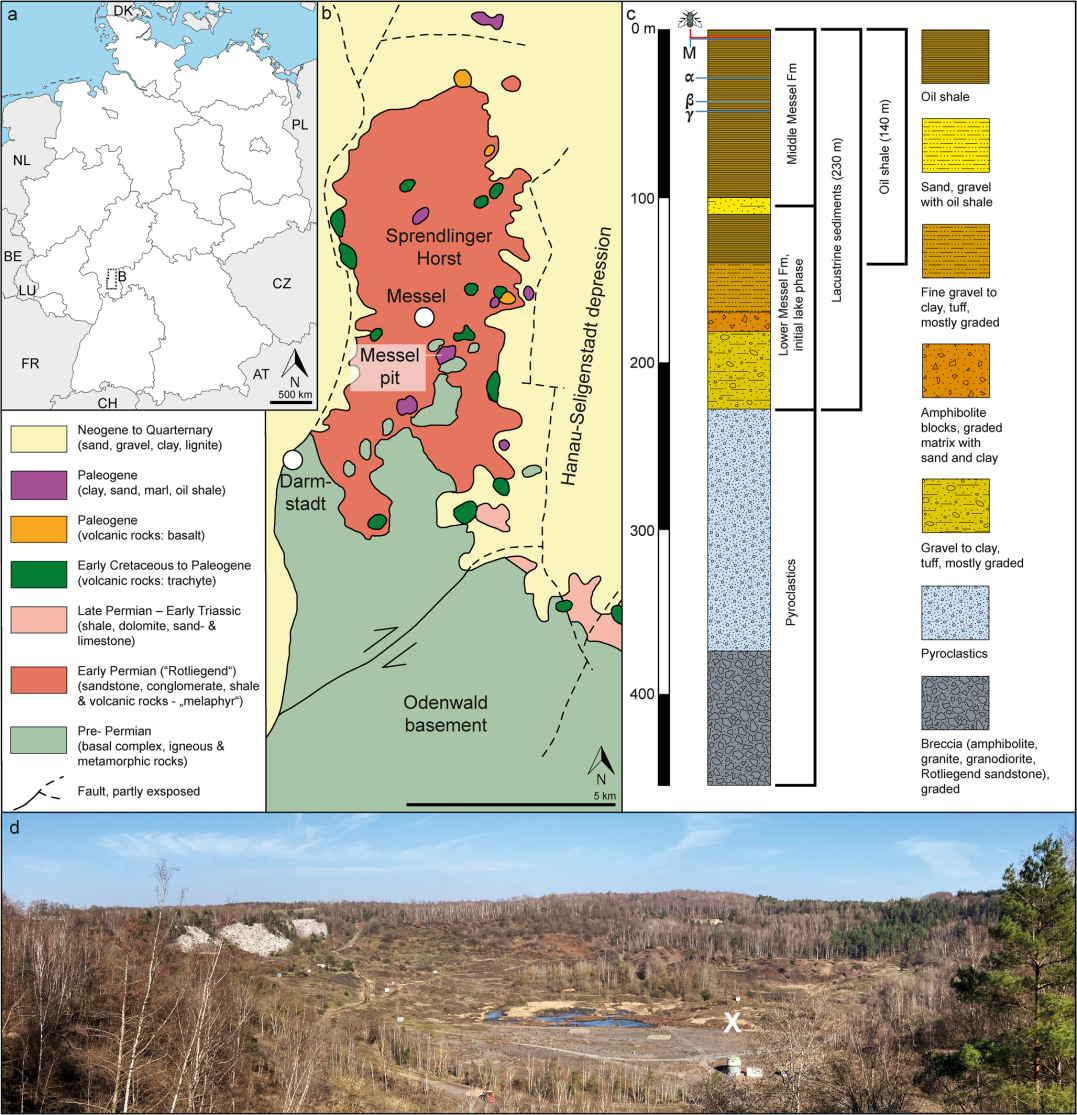
Geographical and geological maps of the Messel pit. **a** Location of the Messel pit in Germany. **b** Simplified geological map of the area surrounding the Messel pit (modified after Harms et al. 1999). **c** Simplified sedimentary profile based on the Messel 2001 drill core (modified after Felder and Harms 2004). The fossil bee was collected 37 cm above local stratigraphic marker level M. **d** Panoramic overview of the Messel pit, X marks the site (grid square H13/14) where the bee was collected

The Messel pit is located on the eastern side of the Rhine Rift Valley, about 8 km northeast of Darmstadt, Germany (Figure 1). The lacustrine sediments of the Messel Formation were deposited within a maar volcanic crater, which had a diameter of at least 1.5 km and an initial depth of 300–400 m (Harms et al. 2003; Felder and Harms 2004; Büchel and Schaal 2018). The sedimentary rocks have been dated as early middle Eocene (early Geiseltalian), with a radiometric age (Ar^40/39^) determination of 47.8 ± 0.2 Ma from volcaniclastic sediments (Mertz and Renne 2005) and 48.27–48.11 Ma ± 0.22 Ma from high-resolution palynological analyses (Lenz et al. 2015). Current estimates based on astronomical tuning date the origin of sediment deposition to have occurred 48.06 million years ago (Kaboth-Bahr et al. 2024). The fossils represent a diverse biota of exceptionally preserved microorganisms, plant organs, insects, fishes, amphibians, reptiles, birds, and mammals (e.g. Gruber and Micklich 2007; Wappler et al. 2015; Smith et al. 2018; Wedmann 2018; Wilde 2018), inferred to represent a paratropical Eocene rainforest (Grein et al. 2011). The stratigraphy of the Messel pit (Figure 1) has been briefly summarised in Wappler and Engel (2003).

The studied bee fossil is preserved in the collection by immersing the slab of oil shale in glycerine to prevent damage by desiccation (Ackermann et al. 1992). The described individual was observed and digitized using a Keyence VHX-7000 microscope, and all relevant structures were measured from the digitized images. All photographs were optimised using Adobe Lightroom CC. Drawings were made from the photographs using Affinity Designer (affinity.serif.com).

The fossil bee was examined and sampled for pollen according to the method described in Grímsson et al. (2021). A stereomicroscope equipped with epifluorescence illumination was used to examine and photograph the fossil. Pollen grains were extracted from the head, metasoma, and legs of the bee and processed for combined light microscopy (LM) and scanning electron microscopy (SEM) analysis by bleaching (Geier et al. 2023c) followed by acetolysis (Halbritter et al. 2018). Pollen grains were investigated using the “single-grain method” by Zetter (1989). Morphological terminology for the description of the bee follows Engel (2001b) and Michener (2007), and the classification of *Xylocopa* follows Michener (2007). The classification of plants follows that of the APG IV (2016), with pollen of Theaceae described first, followed by the Araliaceae. Pollen descriptions include all features observed with both LM and SEM. The pollen terminology follows Punt et al. (2007, LM) and Halbritter et al. (2018, LM, SEM). Taxonomic actions made herein are registered in ZooBank (www.zoobank.org) with the article LSID: urn:lsid:zoobank.org:pub:3B66630B-F719-4A16-ABC6-FF2DB08907CE

## Systematic palaeontology

Kingdom Animalia Linnaeus, 1735

Phylum Arthropoda Gravenhorst, Siebold, 1848

Class Insecta Linnaeus, 1758

Order Hymenoptera Linnaeus, 1758

Family Apidae Latreille, 1802

Tribe Xylocopini Latreille, 1802

Genus *Xylocopa* Latreille, 1802

*Apocolyx* Engel subgen. nov.

**Type species (*hic designatus*):** *Xylocopa* (*Apocolyx*) *primigenia* Engel and Wappler sp. nov.

**Diagnosis:** It is challenging to compare the current fossil with modern relatives in many characters traditionally employed in the classification of *Xylocopa* (e.g. facial carinae, form of metabasitibial plate, sternal and tergal graduli, pygidial plate). Nonetheless, there are sufficient details to make some important distinctions, most notably in the forewing venation. The new subgenus differs from all other groups of *Xylocopa* by the much shorter and broader marginal cell that tapers away from the anterior wing margin near its base and ends at about the apex of the third submarginal cell, rather than that of the living subgenera in which the cell is noticeably slender, elongate, extends well beyond the third submarginal cell, and tapers away from the anterior wing margin in its apical third or quarter. In addition, the second submarginal cell, while having the usual asymmetrical trapezoidal shape of most *Xylocopa* species, is quite exaggerated in its proximal extension and narrow anterior margin, with the posterior margin over 10× greater than the anterior margin (this is usually over 2× in most extant *Xylocopa* but never exceeds 4.5× the length). Additional features of the subgenus include: 2rs-m prominently bowed apically (as in most extant subgenera); given the orientation of the longitudinal axis of the compound eye relative to the vertex it would seem that the eyes do not converge above or below, as in the subgenera *Xylocopoda* Hurd and Moure and some species of *Xylomelissa* Hurd and Moure (*sensu* Michener 2007) (Hurd and Moure 1963); the mesal margin of the compound eye is weakly incurved, as in many subgenera of *Xylocopa*; the vertex is straight before arching laterally above the compound eye, as is the case in various subgenera; the median ocellus is slightly smaller than the antennal torulus, as is the case in most Old World subgenera; the mesoscutellum is not flattened nor projecting over the metanotum.

It would be easy to consider the species reported herein as generically distinct from *Xylocopa* owing to the unique marginal cell and more exaggerated second submarginal cell, but given the greater number of similarities to modern large carpenter bees (e.g. elongate metabasitarsus, trapezoidal second submarginal cell, virtually absent pterostigma, proximally constricted marginal cell, large body size, papillate wing membrane beyond veins) we have conservatively considered the fossil to belong to a putatively early-diverging subgenus.

**Etymology:** The new genus-group name is an anadrome of *Xylocopa* Latreille. The gender of the name is considered to be feminine. The subgeneric name is registered under ZooBank LSID urn:lsid:zoobank.org:act:0BBDAFBE-130F-4EC4-8E5E-8B8E49DC27A7.

*Xylocopa* (*Apocolyx*) *primigenia* Engel and Wappler sp. nov.

(Figure 2)

**Fig. 2:**
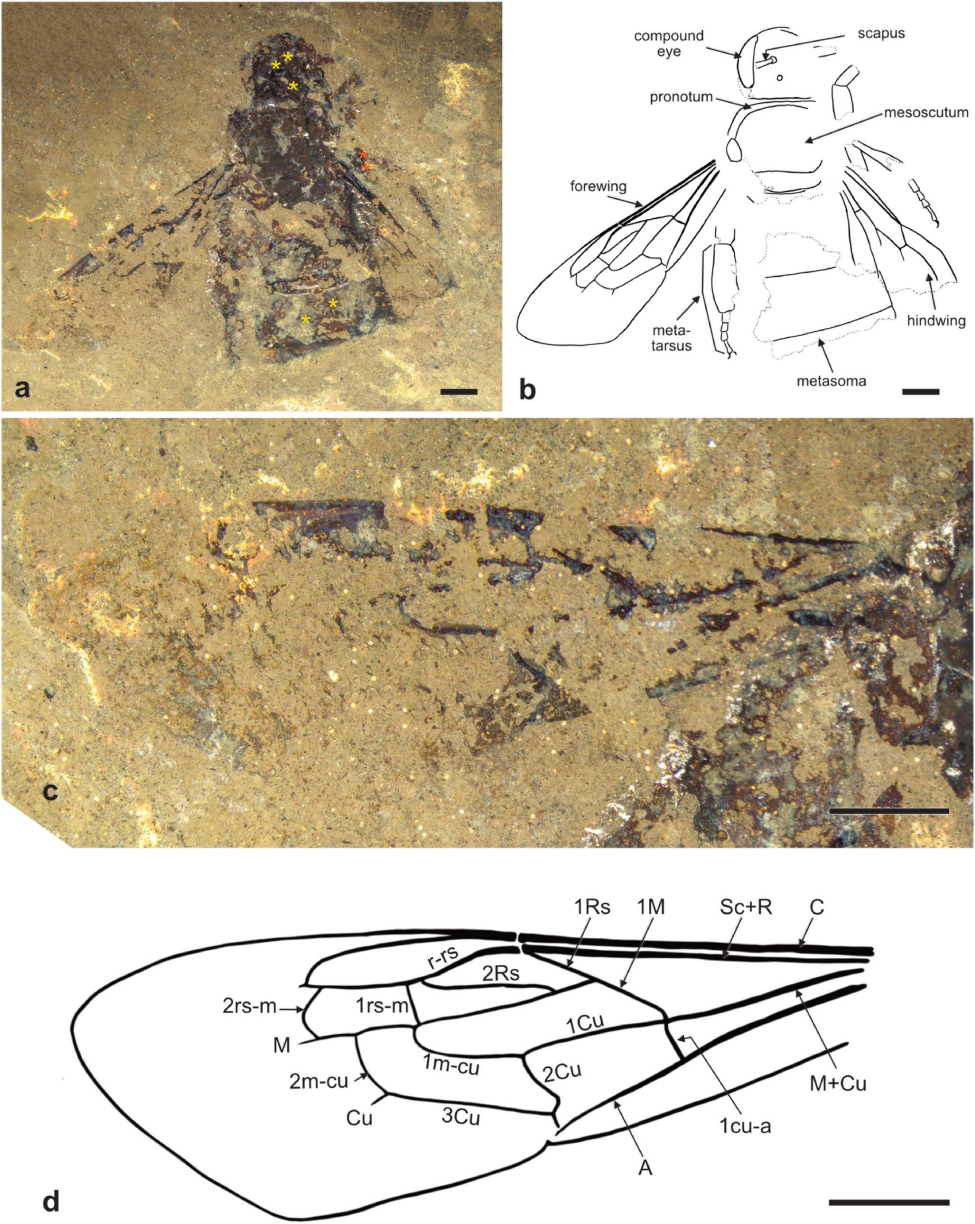
Messel large carpenter bee, *Xylocopa* (*Apocolyx*) *primigenia* subgen. et sp. nov., holotype female. **a, b** Photograph of entire specimen and line drawing of pertinent sclerites; **c, d** Photograph of forewing and line drawing of preserved and reconstructed venation. *Scales* 2 mm; *yellow asterisk* Theaceae pollen extraction area; *orange asterisk* Araliaceae pollen extraction area

**Diagnosis:** As for the subgenus (*vide supra*).

**Description:** ♀: Total body length (as preserved, apical metasomal segments missing so length in life would have been much longer) 17.68 mm; forewing length ~15.08 mm, maximum width 4.66 mm; integument dark brown to black as preserved, sculpturing not discernible as preserved. Head broader than long, medial length 3.92 mm, width (as preserved) 3.99 mm; compound eye length 2.94 mm, mesal margin of compound eye weakly incurved; vertex straight. Mesosoma length 5.62 mm (as preserved, apical portion of propodeum missing), maximum width 5.34 mm; mesoscutum length 3.66 mm, mesoscutellum length 1.11 mm; mesoscutellum not flattened nor extending posteriorly over metanotum (i.e. not as in *Koptortosoma* Gribodo or *Mesotrichia* Westwood), seemingly low and slightly rounded. Forewing prestigma virtually absent; pterostigma virtually absent owing to absence of abscissa of R between r-rs and anterior wing margin; r-rs elongate, angling posteriorly from apex of R to meet 2Rs; R nearly immediately diverging from anterior wing margin (i.e. in proximal portion of marginal cell) to point near tangent with apex of third submarginal cell, then steeply angling posteriorly to meet Rs; apex of marginal cell feebly appendiculate; marginal cell comparatively broad, nearly as wide as third submarginal cell (presumed plesiomorphy; in extant subgenera apical portion of marginal cell 0.5× width or less of third submarginal cell), marginal cell not extending well beyond apex of third submarginal cell (unique apomorphy; marginal cell extending well beyond apex of third submarginal cell in extant subgenera); 1Rs and 1M forming a straight line; 1M nearly confluent with 1cu-a; 1cu-a orthogonal to M+Cu and A; Rs+M comparatively short, slightly less than one-third length of 2M; 2Rs elongate, meeting r-rs close to 1rs-m; 1rs-m straight, approximately orthogonal to Rs, slightly basad 1m-cu; second submarginal cell strongly asymmetrically trapezoidal, with posterior margin more than 10× anterior margin (unique apomorphy); 1m-cu and 2m-cu both entering third submarginal cell, former near base of cell, latter slightly distad cell midlength; third submarginal cell large, much longer than wide; 2rs-m strongly bowed outward; 2Cu with enigmatic posterior bend before origin of 2m-cu (unclear whether this may be an artifact); wing membrane beyond veins papillate. Hind wing with 1Rs straight, 2Rs elongate and weakly arched; rs-m slightly less than 0.5× length 1Rs; 1M slightly longer than 1Rs, slightly more than 2× as long as 2M; 2M comparatively short, slightly longer than rs-m; 1Cu shorter than 1M; 2M+Cu about as long as 1M. Metabasitarsus elongate (as is typical for *Xylocopa*), distinctly longer than combined lengths of remaining tarsomeres and probably longer than metatibia, length 3.75 mm, densely setose. Metasoma broad, wider than mesosoma, width 7.72 mm (as preserved across tergum II).

♂: *Latet*.

**Holotype (*hic designatus*):** ♀, HLMD-Me-15783, deposited in the Hessisches Landesmuseum, Darmstadt, Germany.

**Type locality and horizon:** Messel pit (latitude 49°55′N, longitude 8°45′E) near Darmstadt, Hesse, Germany; Messel Formation, lower mid-Eocene, Geiseltalian, ca. 48 Ma. The specimen was collected in 1997 in grid square H13/14, in strata 37 cm above local stratigraphic marker level M.

**Etymology:** The specific epithet is the Latin adjective *primigenius*, meaning, “firstborn”, “original”, or “primitive”. The specific epithet is registered under ZooBank LSID urn:lsid:zoobank.org:act:80EECF13-F03C-4873-AC1B-C350A433FB95

Kingdom Plantae Haeckel, 1866

Angiospermae

Order Ericales Bercht. and J.Presl, 1820

Family Theaceae Mirb., 1816

Theaceae gen. et sp. indet.

(Figure 3a–l)

**Fig. 3:**
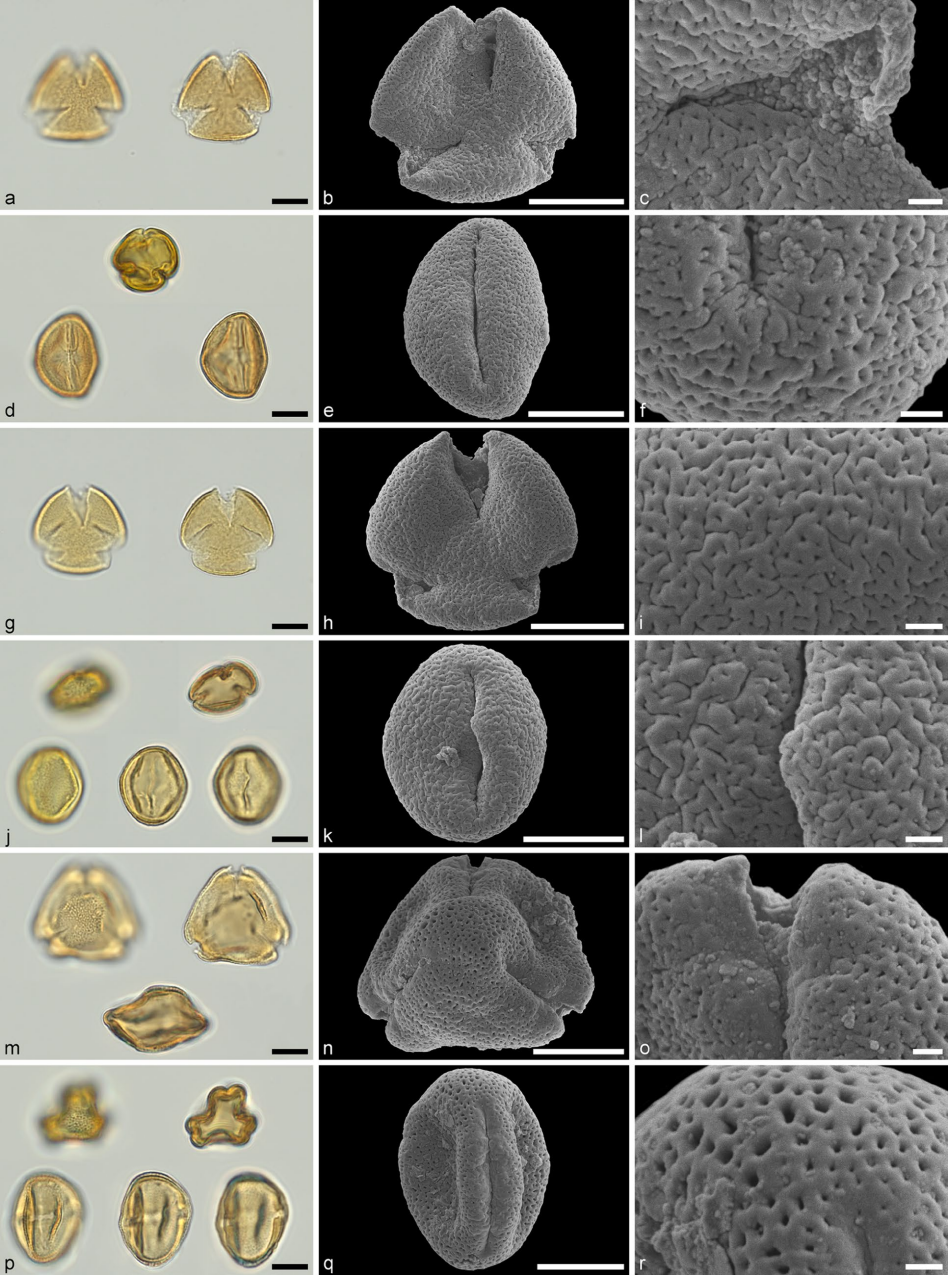
Light microscopy (LM) (**a, d, g, j, m, p**) and scanning electron microscopy (SEM) (**b, c, e, f, h, i, k, l, n, o, q, r**) micrographs of fossil Theaceae and Araliaceae pollen extracted from the fossil carpenter bee (*Xylocopa primigenia* sp. nov.). **a–l** Theaceae pollen extracted from the head (**a–f**) and the metasoma (**g–l**); **m-r** Araliaceae pollen extracted from the mid leg; **a** Theaceae pollen in polar view; **b** same gain as in a; **c** detail of colpus membrane; **d** Theaceae pollen in polar and equatorial view; **e** same grain as in e; **f** detail of polar area and adjacent colpus; **g** Theaceae pollen in polar view; **h** same gain as in g; **i** detail of mesocolpium; **j** Theaceae pollen in polar view and equatorial view; **k** same grain as in j; **l** detail of the bridge covering the porus; **m** Araliaceae pollen in polar view and equatorial view; **n** same grain as in m; **o** detail of the aperture; **p** Araliaceae pollen in polar view and equatorial view; **q** same grain as in p; **r** detail of polar area. Scales = 10 µm (**a, b, d, e, g, h, j, k, m, n, p, q**), 1 µm (**c, f, i, l, o, r**)

**Note:** Pollen extracted from head and metasoma (Figure 2a)

**Description:** Pollen, monad, prolate, outline convex-triangular to circular in polar view, elliptical in equatorial view; polar axis 20–24 µm in LM, 17–22 µm in SEM, equatorial diameter 15–24 µm in LM, 15–21 µm in SEM; tricolporate, endoporus lalongate and weakly outlined, colpi 3/4 of polar axis long (SEM), nexine thickened along colpi towards endoapertures, colpus membrane granulate to microareolate, bridge over porus; exine 1.1–1.7 µm thick (LM), sexine and nexine equally thick; sculpture scabrate in LM, microreticulate, rugulate, fossulate, and perforate in SEM, occasional granulate suprasculture (SEM).

**Remarks:** The current circumscription of Theaceae comprises approximately 367 species in eight genera (Table 1; POWO 2023). To our knowledge there currently exists no comprehensive palynological work on pollen from extant Theaceae and only a small portion of the species have been investigated palynologically. Still, selective taxa from all genera have been published, either using only LM or SEM, combined LM and SEM, or combined SEM and TEM (e.g. IBSCIB-CAS 1982; Wang et al. 1995; Wei 1997; Kataoka et al. 2001; Heo et al. 2011; Li et al. 2011; Miyoshi et al. 2011, and references therein). Based on the combined features observed in the fossil pollen grains we are able to assign this pollen type to Theaceae, but not with certainty to any extant genus. The general size, outline and shape, as well as the aperture configuration in LM is similar between pollen of extant genera. The thickening of the nexine around the apertures observed with LM, as observed in the fossil pollen, is prominent in some species of *Camellia* L. (e.g. Fujiki and Ozawa 2007). The sculpture of the fossil pollen observed with SEM is most comparable to that of pollen from extant *Camellia*, *Schima* Reinw. ex Blume, and *Stewartia* L. (e.g. Wei 1997; Kataoka et al. 2001; Fujiki and Ozawa 2007; Heo et al. 2011). Based on the available information from pollen morphology and ultrastructure of extant Theaceae it is most conservative to state that the fossil pollen is an morphological intermediate type between the three aforementioned genera and could be affiliated with any of them, but see fossil record below.

**Table 1:** Numbers of extant genera and species of Theaceae and Araliaceae. Note: Generic names and species numbers compiled from POWO (2023)

**Theaceae**	**Species**	**Araliaceae**	**Species**
*Apterosperma* Hung T.Chang	1	*Hedera* L.	19
*Camellia* L.	230	*Heptaleurum* Gaertn.	321
*Franklinia* W. Bartram ex Marshall	1	*Heteropanax* Seem.	9
*Gordonia* Ellis	22	*Hydrocotyle* Tourn. ex L.	177
*Polyspora* Sweet	49	*Kalopanax* Miq.	1
*Pyrenaria* Blume	27	*Macropanax* Miq.	18
*Schima* Reinw. ex Blume	16	*Merrilliopanax* H.L.Li	3
*Stewartia* L.	22	*Meryta* J.R.Forst. and G. Forst.	27
		*Metapanax* J.Wen and Frodin	2
**Araliaceae**	**Species**	*Motherwellia* F.Muell.	1
*Anakasia* Philipson	1	*Neocussonia* Hutch.	16
*Aralia* L.	73	*Neopanax* Allan	5
*Astropanax* Seem	15	*Oplopanax* (Torr. and A.Gray) Miq.	3
*Astrotricha* DC.	20	*Oreopanax* Decne. and Planch.	146
*Brassaiopsis* Decne. and Planch.	46	*Osmoxylon* Miq.	61
*Cephalaralia* Harms	1	*Panax* L.	14
*Cephalopanax* G.M.Plunkett, Lowry & D.A.Neill	2	*Plerandra* A. Gray	33
*Cheirodendron* Nutt. ex Seem	6	*Polyscias* J.R. and G.Frost	180
*Chengiopanax* C.B.Shang & J.Yhuang	2	*Pseudopanax* K.Koch	7
*Crepinella* Marchal	33	*Raukaua* Seem.	6
*Cussonia* Thinb.	20	*Schefflera* J.R.Frost. and G.Frost.	13
*Dendropanax* Decne. & Planch.	95	*Sciodaphyllum* P.Browne	145
*Didymopanax* Decne. & Planch.	38	*Sinopanax* H.L.Li	1
*Eleutherococcus* Maxim.	29	*Tetrapanax* (K.Koch) K.Koch	1
*Fatsia* Decne. & Planch.	3	*Trachymene* Rudge	59
*Frodinia* Lowry & G.M.Plunkett	2	*Trevesia* Vis.	8
*Gamblea* C.B.Clarke	4	*Seemannaralia* R.Vig.	1
*Harmsiopanax* Warp.	3	*Woodburnia* Prain	1

Order Apiales Nakai, 1930

Family Araliaceae Juss., 1789

Araliaceae gen. et sp. indet.

(Figure 3m–r)

**Note:** Pollen extracted from mid leg (Figure 2a)

**Description:** Pollen, monad, prolate, outline triangular to tri-lobate in polar view, lens-shaped to elliptic in equatorial view; polar axis 19–31 µm in LM, 23–27 µm in SEM, equatorial diameter 16–30 µm in LM, 17–25 µm in SEM; tricolporate, endoporus large and lalongate, colpus 2/3 of polar axis (SEM), massive nexine thickening along apertures (up to 3 µm thick in LM); exine 1.1–1.8 µm thick (LM), sexine as thick or thicker than nexine; sculpture reticulate in LM and SEM, reticulum gradually decreasing in size from polar areas and mesocolpium towards colpi, forming a perforated and psilate margin along colpi (SEM).

**Remarks:** Araliaceae are a large family, currently comprising about 1680 species in 46 genera (Table 1; POWO 2023). There are several publications on the pollen morphology (LM, SEM) and ultrastructure (TEM) of Araliaceae pollen (e.g. Tseng 1971; Huang 1972; Tseng and Shoup 1978; Tseng et al. 1983; Henwood 1991; Wang et al. 1995; Wen and Nowicke 1999; Fiaschi et al. 2010; Miyoshi et al. 2011; Wildner 2017, and references therein). Based on the combined morphological traits (incl. size, shape, reticulate sculpture, exine thickness, thickened nexine around apertures, and lalongate endopori) observed with combined LM and SEM we assign this pollen type to the Araliaceae. However, pollen of species and genera of Araliaceae often overlap in most diagnostic morphological LM and SEM based features. Members of *Dendropanax* Decne. and Planch.*, Eleutherococcus* Maxim.*, Heteropanax* Seem.*, Macropanax* Miq.*, Merrilliopanax* H.L.Li*, Metapanax* J.Wen and Frodin*, Neopanax* Allan*, Polyscias* J.R.Forst. and G.Forst., and some of *Schefflera* J.R.Forst. and G.Forst. and *Tetrapanax* (K.Koch) K.Koch are more likely to be the parent plants of the fossil araliaceous pollen. Others, including *Aralia* L., *Didymopanax* Decne. and Planch., *Dendropanax*, *Kalopanax* Miq.*,* and some members of *Panax* L. and *Schefflera* have ambivalent morphologies. Only a few genera, including *Brassaiopsis* Decne. and Planch.*, Fatsia* Decne. and Planch.*, Hedera* L., *Oplopanax* (Torr. and A.Gray) Miq.*, Osmoxylon* Miq.*, Panax* L., and *Trevesia* Vis, exhibit differences in pollen morphology that allow for them to be excluded as the potential parent plant of the fossil araliaceous pollen type. Therefore, this fossil pollen type could consequently belong to several extant genera, but see fossil record below.

## Discussion

### Fossil record of parent plants – match or mismatch with the Messel flora?

The fossil record of Theaceae dates back to the Upper Cretaceous with scarce findings of *Pollenites* pollen (*Gordonia* Ellis and *Schima* type) from Alabama, U.S.A. (Leopold and Pakiser 1964); *Palaeoschima* seeds (*Schima* like) from Germany, Austria, and Czechia (Knobloch and Mai 1986); and *Schimoxylon* wood (similar to *Schima*) from Egypt (Kramer 1974). Most other Cretaceous records have been rejected or are considered doubtful by Grote and Dilcher (1989, 1992). Cenozoic records are numerous, including both extinct genera like *Andrewisocarpon* (Grote and Dilcher 1989) and *Gordoniopsis* (Grote and Dilcher 1992) as well as extant representatives of *Camellia*, *Gordonia*, *Polyspora* Sweet, *Schima*, and *Stewartia* (e.g. Li et al. 2013; Huang et al. 2016; Shi et al. 2017; Erdei and Hably 2021). Even though Paleocene fossils of Theaceae are more or less absent, other Paleogene records, especially of Eocene age, show that the family had already diverged and dispersed across the globe during that time. *Camellia* (and alike) fossils have been reported from the middle Eocene of Germany (fruit: Collinson et al. 2012); the late Eocene of Honshu, Japan (leaves: Huzioka and Takahasi 1972); the early Oligocene of Washington, U.S.A (leaves: Wolfe 1968) and Bulgaria (leaves: Palamarev et al. 2000); and the late Oligocene of Nanning, South China (wood: Huang et al. 2016). Fossils of *Gordonia* are documented from the middle Eocene of Kentucky and Tennessee U.S.A (fruits/seeds: Grote and Dilcher 1992); the middle to late Eocene of Germany (leaves: Mai and Walther 1985, 2000); the late Eocene of England (fruits: Chandler 1926, 1961); and the Oligocene of Japan (leaves: Tanai 1970). Fossils of *Polyspora* have been reported from the middle to late Eocene of Germany (leaves: Kvaček and Walther 1984; Mai and Walther 1985, 2000; Wilde 1989) and the late Eocene of Czechia (leaves: Kvaček and Walther 1984). *Schima* (and alike) fossils are known from the middle Eocene of Germany (fruits: Mai 1971), the middle Eocene of Myanmar (wood: Licht et al. 2014), and the late Oligocene of Guangxi, China (fruits: Shi et al. 2017). The Theaceae pollen type reported herein is not the first representative of this family from the Messel pit. Wilde (1989) described from this locality leaves of *Polyspora* and Collinson et al. (2012) fruit of *Camelliacarpoidea* (similar to *Camellia*). Considering these two records as well as other Eocene fossils of Theaceae reported from Germany (e.g. Mai 1971; Kvaček and Walther 1984; Mai and Walther 1985, 2000), it is likely that the fossil theaceous pollen belongs to one of the following genera: *Camellia*, *Gordonia*, *Polyspora*, or *Schima*.

The early fossil pollen records of Araliaceae extend back to the Upper Cretaceous (lower Campanian) of Wyoming, U.S.A.; Upper Cretaceous (Senonian) of China; the Paleocene and Eocene of Greenland; the Eocene of western North America; and the Paleocene (France) and Eocene (Germany) of Europe (Muller 1981; Song et al. 2004; Manchester et al. 2015). Early macrofossil records include fruit/seeds of *Acanthopanax* (Decne. and Planch.) Witte (now within: *Eleutherococcus* Maxim.) and *Aralia* from the Upper Cretaceous (Maastrichtian) of Germany (Knobloch and Mai 1986), flowers of *Tetraplasandranthus* from the Upper Cretaceous/Eocene of India (Kapgate et al. 2009), araliaceous wood (*Plerandreoxylon*), fruits (*Paleopanax*; similar to *Pseudopanax*), and leaves from the middle Eocene of Oregon, U.S.A. (Manchester 1994; Wheeler and Manchester 2002), and leaves of *Dendropanax* from the middle Eocene of Tennessee (Dilcher and Dolph 1970). As with the Theaceae, the fossil record of Araliaceae suggests that the family had already dispersed around the globe prior to the end-Eocene and was represented by a number of extinct and extant genera by that time. Oligocene records include mostly fossils assigned to extant genera, such as *Aralia* (now incl. *Pentapanax* Seem.) and *Schefflera* from Germany (Mai and Walther 1991; Mai 1997; Bozukov 2018). Fossils of Araliaceae from Messel are rare and restricted to cuticle remains of cf. *Fatsia* (Wilde 1989) and two *Araliaceoipollenites* pollen taxa (Thiele-Pfeiffer 1988). Dispersed pollen assigned to the larger subspecies of *A. euphorii* from Messel (plate 16, fig. 1–4 in Thiele-Pfeiffer 1988) is probably the same pollen type (shared parent plant/taxon) as the Araliaceae pollen extracted from the fossil bee. As to date, no flower containing this pollen type is described from Messel, leaving a more precise affiliation pending. Considering the morphology of the fossil pollen and extant Araliaceae genera that have been documented in Europe in the Eocene-Oligocene, it is possible that the araliaceous pollen type from the bee originates from *Schefflera*.

### Pollination ecology of Theaceae and Araliaceae

Knowledge about the pollination biology of Theaceae is meagre and only a few species have been investigated thoroughly. Among all genera, *Camellia* is the most studied due to its economic importance for both tea and oil production (Stevens et al. 2004). *Camellia sinensis* (L.) Kuntze shows floral morphologies of a generalist pollination syndrome and is visited and pollinated by various animals including short-tongued flies, bees and wasps, beetles, moths, butterflies, and true bugs (Wickramaratne and Vitarana 1985). For *C. pubipetala* Y.Wan and S.Z.Huang the primary pollinators are sun birds (*Aethopyga christinae* Swinhoe) and honey bees (*Apis cerana* Fabricius). During nectar feeding both sunbirds and bees come into contact with male and female reproductive organs of the flowers and are covered with pollen. Observations show that birds have pollen on their beaks and heads, while bees carry pollen on their entire body including mouthparts, metasoma, and legs (Chai et al. 2019). Flowers of *C. oleifera* C.Abel are also visited by a number of insects, especially bees and flies but also wasps. Bee visitors include cultivated honey bees (*A. cerana* and *A. mellifera* L.) as well as wild bees (*Colletes gigas* Cockerell*, Andrena striata* Fabricius*, A. camellia* Wu*, A. hunanensis* Wu*, A. chekiangensis* Wu). Flies that visit flowers of *C. oleifera* belong to the Calliphoridae, Muscidae, Sarcophagidae, and especially hover flies (family Syrphidae). The only wasp observed visiting *C. oleifera* flowers was *Vespa velutina* Lepeletier (Li et al. 2021; Yuan et al. 2022). *Camellia japonica* L. is also visited by song birds (among others, *Zosterops erythropleura erythropleura* Swinhoe), honey bees (*A. cerana* and *A. mellifera*), syrphid hover flies, and even a squirrel has been observed visiting the flowers (Yumoto 1987; Rho and Choe 2003). Flowers of *C. sasanqua* Thunb. are visited by birds, wasps, syrphid flies, moths, butterflies, and colletid bees (Yumoto 1987). *Gordonia lasianthus* (L.) Ellis is visited by small beetles, ants, and halictid bees (Brown 2019), while flowers of *Schima superba* Gardner and Champ. are visited by *A. cerana* and scarab beetles (*Protaetia brevitarsis* (Lewis) and *Popillia mutans* (Newman)) (Yang et al. 2017). *Schima wallichii* (DC.) Korth. primary pollinators are honey bees (*Apis* L.), carpenter bees (*Xylocopa*), and butterflies (*Sinthusa nasaka* (Horsfield)*, Cabera pusaria* (L.), and *Dysphania militaris* (L.)) (Khanduri 2001; Khanduri et al. 2013). Based on the currently available literature on flower visitations and pollinators of Theaceae it is clear that the family is animal pollinated and insects are the most common pollinators. Bees and flies seem to be the most frequent visitors/pollinators, followed by beetles and butterflies. Extant species of *Xylocopa* are important generalist pollinators in tropical, subtropical, and warm temperate regions and visit species of Theaceae, especially the flowers of *Schima*. *Xylocopa* (*Nyctomelitta*) *tranquebarica* (Fabricius) is known to visit Theaceae, and *X.* (*Biluna*) *nasalis* Westwood collects pollen of *Schima* that is fed to the larvae (Khanduri 2001; Burgett et al. 2005; Khanduri et al. 2013; Hongjamrassilp and Warrit 2014).

Extensive studies on the pollination ecology of Araliaceae are lacking. In general, Araliaceae are pollinated primarily by insects, which are attracted by both nectar and pollen as rewards. Pollinators include flies (Bombyliidae, Calliphoridae, Muscidae, Syrphidae), bees and wasps (Apidae, Formicidae, Halictidae, Ichneumonidae, Vespidae), butterflies (Nymphalidae, Tortricidae), and beetles (Scarabeidae). Bird and bat pollination are also encountered, but are rare (Plunkett et al. 2018). *Aralia hispida* Vent. is probably pollinated by bumble bees (*Bombus vagans* Smith and *B. terricola* Kirby; Thomson and Barrett 1981). The pollinators of *Eleutherococcus trifoliatus* (L.) S.Y.Hu include flies of the families Syrphidae and Tachinidae, as well as honey bees (*Apis*) (Xiao et al. 2020). *Hedera helix* L. is visited by about 20 taxa of insects. Wasps seemed to be important pollinators, next to honey bees, bumble bees, bristle flies, as well as small and large hover flies (Jacobs et al. 2010). Both *Panax quinquefolius* L. and *P. wangianus* S.C.Sun are visited by syrphid flies and halictid bees (Mooney and McGraw 2007; Venugopal and Preeti 2014). Flowers of *Polyscias pancheri* (Baill.) Harms are visited by nomiine bees (*Austronomia sicheli* (Vachal)), wasps (unidentified), scarab beetles (*Heteronyx caledoniae* Fauvel), as well as ants that feed on nectar (*Polyrhachis guerini* Roger) (Schlessman et al. 1990). *Schefflera heptaphylla* Z.H.Tsi floral visitors comprise primarily flies (*Chrysomya* Robineau-Desvoidy and Syrphinae Latreille) and wasps (*Vespula* Thomson and *Eumenes* Latreille) (Pei et al. 2011).

The limited data on floral visitors and pollinators of Theaceae and Araliaceae are certainly consistent with the observation of bee visitation during the Eocene. The genus *Xylocopa* belongs to the same family (Apidae), as honey bees and bumble bees, albeit quite distantly related within that clade, and includes largely polylectic bees, some of whose species are today known to visit at least theaceous flowers, and likely also those of the Araliaceae. It is clear from the areas of localization of pollen on the fossil bee that early large carpenter bees were potential pollinators of flowers for both of these plant families in the local environment of Messel during the Eocene (Peña-Kairath et al. 2023). Today species of *Xylocopa* transport pollen in often small loads on their legs (principally the metatibia and metabasitarsus), moving the pollen to the hind legs from the fore- and midlegs, and therefore with pollen occurring on all legs to some degree. In addition, species of *Xylocopa* are capable of buzz pollination whereby the sonicate flowers to dislodge pollen on to their venter, including the metasoma, and then use the legs to redistribute the pollen to their legs (e.g. Vallejo-Marín 2018). Poricidal anthers necessitating buzz pollination are rare in Theaceae and Araliaceae and it is unlikely the Eocene species necessitated sonication to dislodge pollen. The heads of bees are also often covered with pollen when entering the corolla. Thus, the locations of the pollen on the fossil are consistent with modern species of *Xylocopa* visiting flowers for pollen and nectar (Michener 2007). Since we discovered pollen from two completely different angiosperm families we assume a polylectic behaviour for the fossil *Xylocopa* bee. The family Apidae is well represented at Messel, with various species of the extinct genera *Electrapis* Cockerell and *Protobombus* Cockerell (Wappler and Engel 2003), both of which were also found to have visited a variety of flowers, including Araliaceae from Messel (Wappler et al. 2015; Grímsson et al. 2017). However, it is possible that some were oiligolectic as suggested by the eucerine bee (Eucerini; a tribe with numerous oligolectic species) record from Messel (Wappler and Engel 2003). Unfortunately, it is not yet possible to make a comparison of pollen collection by fossil xylocopine bees as *X. primigenia* is the first extinct species of its subfamily to have been documented with adhering pollen. Fossils of Xylocopinae are rare (Michez et al. 2012) and most were described long ago necessitating the location of historical type specimens to explore for potential pollen. For at least those species of *Xylocopa* described from the mid-Miocene of Öhningen, Germany no pollen was located on material preserved at ETH Zürich, Switzerland (M.S.E. pers. obs.). Only through greater sampling of potential pollinating insects from Messel and documentation of their associated pollen will permit a fuller understanding of the ecology of this ancient ecosystem. Fortunately, the exceptional preservation of fossils at Messel makes this an ideal locality from which to build such a comprehensive picture of flower-pollinator interrelationships from the Eocene.

## Data Availability

All data generated or analysed during this study are included in this published article. The holotype of *Xylocopa* (*Apocolyx*) *primigenia* Engel and Wappler sp. nov. is deposited in the Hessisches Landesmuseum, Darmstadt, Germany (HLMD-Me-15783).
